# Workflow and sedation choice with the PFA variable-loop circular catheter in real-world AF procedures: insights from the prospective multi-centre VARIPURE clinical study

**DOI:** 10.1093/europace/euag025

**Published:** 2026-02-08

**Authors:** Philipp Sommer, Mads B Kronborg, Frédéric Sebag, Christian Sohns, Tom De Potter, Francis Bessière, Pedro Adragão, Carlo Pappone, Daniel Scherr, Mattias Duytschaever, Alexandre Almorad

**Affiliations:** Department of Electrophysiology, Heart and Diabetes Centre NRW, Ruhr University Bochum, Georgstr. 11, 32545 Bad Oeynhausen, Germany; Department of Cardiology, Aarhus University Hospital, Aarhus, Denmark; Unit of Cardiac Surgery and Department of Cardiology, Institute Mutualiste Montsouris, Paris, France; Department of Electrophysiology, Heart and Diabetes Centre NRW, Ruhr University Bochum, Georgstr. 11, 32545 Bad Oeynhausen, Germany; AZORG, Cardiovascular Centre, Aalst, Belgium; Cardiac Arrhythmia Department, Institut de Cardiologie des Hospices Civils de Lyon, France; Cardiology and Electrophysiology Department, Hospital de Santa Cruz Lisbon, Portugal; Department of Arrythmology, Istituto di Ricovero e Cura a Carattere Scientifico Policlinico San Donato, Milan, Italy; Division of Cardiology, Medical University of Graz, Graz, Austria; Department of Cardiology, AZ Sint-Jan, Brugge, Belgium; Heart Rhythm Management Centre, Postgraduate Program in Cardiac Electrophysiology and Pacing, Universitair Ziekenhuis Brussels – Vrije Universiteit Brussels, European Reference Networks Guard-Heart, Brussels, Belgium

**Keywords:** Variable-loop circular catheter, VARIPULSE, Pulsed field ablation, Workflow, Sedation, Atrial fibrillation

Pulsed field ablation (PFA) has emerged as a promising technology to treat cardiac arrhythmias, including atrial fibrillation (AF).^1–4^ The initial safety and effectiveness of the VARIPULSE Platform [VARIPULSE variable-loop circular catheter (VLCC) and TRUPULSE generator compatible with an electroanatomical mapping system, CARTO 3; Biosense Webster, Inc., part of Johnson & Johnson MedTech] were evaluated in 2 clinical studies restricted to narrowly defined paroxysmal AF populations.^5,6^ VARIPURE, a single-arm, multi-centre, prospective, non-randomized, observational substudy of the SECURE post-market follow-up study (ClinicalTrials.gov Identifier: NCT04750798) was created to address the need for evaluating the real-world safety and performance of the VLCC in contemporary clinical practice. Here, we report ablation workflows and procedural efficiency of PFA ablations performed in a prospectively enrolled patient cohort in Europe, the Middle East, and Africa (EMEA).

Study data were acquired through an electronic data capture system with meticulous monitoring, cleaning, and safety review to preserve the integrity and accuracy of the dataset. Additionally, CARTONET’s artificial intelligence–driven workflow analytics were used for semiautomatic anatomic ablation annotations, facilitating the investigators’ detailed analysis of ablation sequence, timing parameters, and procedural adherence to standardized and optimal workflow guidelines. The CARTONET algorithm automatically annotated lesion anatomical locations, which were centrally reviewed for accuracy. There were no operator-adjusted lesion annotations, eliminating potential site variability in CARTONET-derived data. As this was an observational post-market study, the handling of atypical anatomies was left to the discretion of each operator. Institutional review board approval was obtained in all participating sites, and patients were required to provide written informed consent prior to enrolment. The data were summarized using descriptive statistics. No inferential statistical tests were conducted.

Between April 2024 and June 2025, 791 patients with AF [mean ± SD age, 64.8 ± 10.6 years; male, 62.1%; median (95% CI) CHA_2_DS_2_-VASc, 2.0 (1.0–3.0); paroxysmal AF, 62.1%] underwent *de novo* pulmonary vein isolation (PVI) across 20 EMEA centres performed by 62 operators. Of these, 553 procedures (69.9%) involved PVI exclusively within the pulmonary veins (PVs), while 46 cases (5.8%) included additional right atrial substrate ablations. Nearly one-quarter (24.3%; 192/791) received additional left atrial substrate (LAS) ablations. Among PVI + LAS cases, the most common additional lesion sets targeted the posterior wall (72.9%), roof (53.6%), or anterior wall (24.0%).

Vagal response was defined as bradycardia < 40 beats per minute, asystole, or atrioventricular block. They occurred in 93 patients (11.8%) across the overall population, most frequently during ablation of the left superior PV (6.2%) compared with the other 3 veins (1.9–3.4%), consistent with findings reported for other PFA technologies.^7^ Among 368 procedures without prophylactic measures, 59 (16.0%) exhibited a vagal response. In contrast, preventive strategies were implemented in 413 patients, of whom 31 (7.5%) experienced a vagal response. Atropine was used preventively in 374 cases, with only 6.4% (24/374) developing a vagal response, and atropine timing was per standard of care. These results demonstrate the protective effect of pre-emptive atropine administration in mitigating PFA-associated vagal reactions.

An analysis of 621 procedures with CARTONET data revealed that 75.8% of cases were started on the left-sided vein pairs, with the left superior PV targeted for first ablations in 59.9% of total cases. Procedure time, left atrial (LA) dwell time, fluoroscopy time, and ablation duration were comparable regardless of the side/region ablated first (*Figure* *1Ax* and *B*). There was high adherence to the optimal workflow outlined in the device’s instructions for use, with 90.5% of cases with 4-vein anatomy (*n* = 580) following the recommended 16 to 28 valid ablations for PVI, which indicate reproducible operator performance and adherence to standardized ablation parameters across centres.

**Figure 1:**
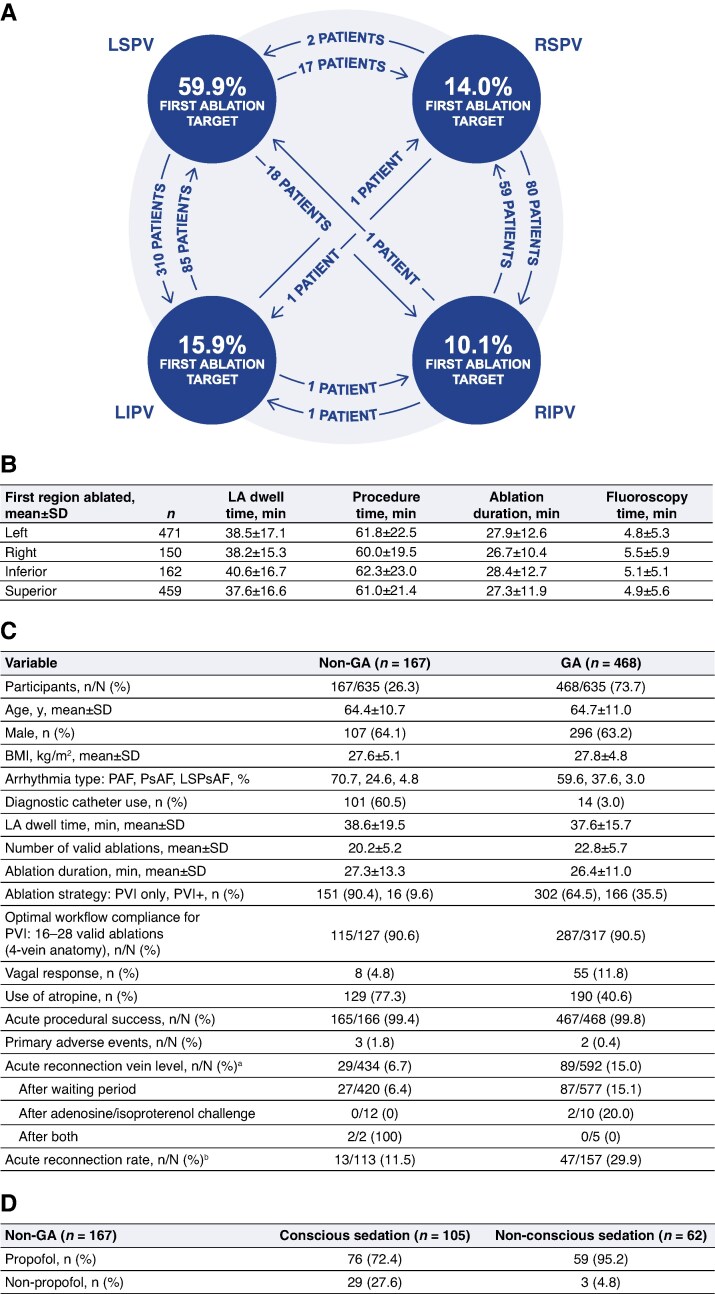
(*A*) Analysis of transitions between the first and second targeted veins and (*B*) key procedural metrics (*n* = 621). (*C*) Sedation subanalysis results (*n* = 635). (*D*) Non-GA (*n* = 167). BMI, body mass index; GA, general anaesthesia; LA, left atrial; LIPV, left inferior pulmonary vein; LSPsAF, long-standing persistent atrial fibrillation; LSPV, left superior pulmonary vein; PAF, paroxysmal atrial fibrillation; PsAF, persistent atrial fibrillation; PV, pulmonary vein; PVI, pulmonary vein isolation; PVI+, pulmonary vein isolation and ablations outside the pulmonary veins; RIPV, right inferior pulmonary vein; RSPV, right superior pulmonary vein. ^a^*N* is the number of PVs in patients who underwent waiting period and/or adenosine/isoproterenol challenge with available data. *n* is the number of PVs with reconnection observed in ≥1 PV. ^b^*N* is the number of patients who underwent waiting period and/or adenosine/isoproterenol challenge with available data. *n* is the number of patients with reconnection observed in ≥1 PV. The figure is courtesy of Biosense Webster, Inc., part of Johnson & Johnson MedTech. ©All rights reserved.

Among the 635 patients with available anaesthesia data, 468 (73.7%) underwent ablation under general anaesthesia (GA) and 167 (26.3%) under non-GA protocols (*Figure 1C*). The non-GA group was sedated using conscious (62.9%) and non-conscious (37.1%) sedation methods. Sites reported that propofol was utilized in 72.4% of patients with conscious sedation and 95.2% of patients with non-conscious sedation (*Figure 1D*). Diagnostic catheter use was markedly higher in non-GA cases (60.5% vs. 3.0%), likely reflecting operator and site-specific workflow preferences. LA dwell time, ablation duration, and number of valid ablations were comparable between the non-GA and GA groups (mean ± SD: 38.6 ± 19.5 min vs. 37.6 ± 15.7 min, 27.3 ± 13.3 min vs. 26.4 ± 11.0 min, and 20.2 ± 5.2 vs. 22.8 ± 5.7, respectively). Atropine was used preventively more often in non-GA procedures (77.3% vs. 40.6%), and operators reported a lower incidence of vagal responses with non-GA protocols (4.8% vs. 11.8%). Despite these differences, adherence to the optimal workflow for PVI was similar between groups (90.6% non-GA vs. 90.5% GA). Acute procedural success remained uniformly high (99.4% vs. 99.8%), and primary adverse event rates were low and comparable between groups (1.8% vs. 0.4%; *Figure 1C*). In the non-GA group, 88.5% of patients were free of acute reconnection compared with 70.1% of patients in the GA group; no patients underwent repeat ablation in both groups for the primary arrhythmia post-blanking period (Days 91–365) at the time of data extract. Collectively, these results indicate that the VLCC platform provides procedural efficiency, workflow reproducibility, and safety irrespective of the sedation method.

This analysis of the ongoing, prospective VARIPURE post-market follow-up study demonstrates that the VLCC enables efficient, safe, and reproducible workflows for PFA-based AF ablation in a real-world heterogeneous patient population. Consistent procedural success was achieved despite variability in the operator’s choice of ablation strategy and sedation method. The use of lesion annotation provided valuable insights into workflow patterns and catheter navigation, supporting the VLCC’s versatility and adaptability across diverse clinical settings. LA dwell times for VARIPULSE (GA and non-GA groups) are also comparable to other technologies.^8,9^ Collectively, these results highlight high compliance with workflow standards, high acute success, and uniformly low complication rates across anaesthesia types, emphasizing the robustness of the platform in routine clinical practice. These findings provide a foundation for standardized PFA workflows and highlight the need for continued follow-up to assess the impact of optimal lesion strategies on long-term clinical outcomes.

## Data Availability

Johnson & Johnson MedTech has an agreement with the Yale Open Data Access (YODA) Project to serve as the independent review panel for the evaluation of requests for clinical study reports and patient-level data from investigators and physicians for scientific research that will advance medical knowledge and public health. Requests for access to the study data can be submitted through the YODA Project site at http://yoda.yale.edu.

## References

[1] Bradley CJ, Haines DE. Pulsed field ablation for pulmonary vein isolation in the treatment of atrial fibrillation. J Cardiovasc Electrophysiol 2020;31:2136–47. 32107812 10.1111/jce.14414

[2] Tzeis S, Gerstenfeld EP, Kalman J, Saad EB, Sepehri Shamloo A, Andrade JG, et al 2024 European Heart Rhythm Association/Heart Rhythm Society/Asia Pacific Heart Rhythm Society/Latin American Heart Rhythm Society expert consensus statement on catheter and surgical ablation of atrial fibrillation. Europace 2024;26:euae043.38587017

[3] Chun KJ, Miklavcic D, Vlachos K, Bordignon S, Scherr D, Jais P et al State-of-the-art pulsed field ablation for cardiac arrhythmias: ongoing evolution and future perspective. Europace 2024;26:euae134. 38848447 10.1093/europace/euae134 PMC11160504

[4] Bergonti M, Mills MT, Roten L, Ruwald MH, Metzner A, Conte G, et al Pulsed field ablation for atrial fibrillation ablation: a European Heart Rhythm Association survey. Europace 2025;27:euaf294. 41363248 10.1093/europace/euaf294 PMC12686987

[5] Reddy VY, Calkins H, Mansour M, Wazni O, Di Biase L, Bahu M, et al Pulsed field ablation to treat paroxysmal atrial fibrillation: safety and effectiveness in the AdmIRE Pivotal Trial. Circulation 2024;150:1174–86. 39258362 10.1161/CIRCULATIONAHA.124.070333 PMC11458102

[6] De Potter T, Grimaldi M, Duytschaever M, Anic A, Vijgen J, Neuzil P et al Predictors of success for pulmonary vein isolation with pulsed-field ablation using a variable-loop catheter with 3D mapping integration: complete 12-month outcomes from inspIRE. Circ Arrhythm Electrophysiol 2024;17:e012667. 38655693 10.1161/CIRCEP.123.012667 PMC11111320

[7] Del Monte A, Della Rocca DG, Pannone L, Vetta G, Cespon Fernandez M, Marcon L et al Pulsed field ablation of the right superior pulmonary vein prevents vagal responses via anterior right ganglionated plexus modulation. Heart Rhythm 2024;21:780–7. 38290688 10.1016/j.hrthm.2024.01.040

[8] Boersma LVA, Szeplaki G, Dello Russo A, García-Bolao I, Efremidis M, Szegedi N et al Real-world experience with the pentaspline pulsed field ablation system: one-year outcomes of the FARADISE registry. Europace 2025;27:euaf182. 40888735 10.1093/europace/euaf182 PMC12400809

[9] Metzner A, Fiala M, Vijgen J, Ouss A, Gunawardene M, Hansen J, et al Long-term outcomes of the pentaspline pulsed-field ablation catheter for the treatment of paroxysmal atrial fibrillation: results of the prospective, multicentre FARA-Freedom Study. Europace 2024;26:euae053. 38385529 10.1093/europace/euae053 PMC10932745

